# Dynamics of actin polymerisation during the mammalian single-cell wound healing response

**DOI:** 10.1186/s13104-019-4441-7

**Published:** 2019-07-16

**Authors:** Corina DeKraker, Laurence Goldin-Blais, Eric Boucher, Craig A. Mandato

**Affiliations:** Department of Anatomy and Cell Biology, Faculty of Medicine, McGill University, 3640 University Street, Strathcona Anatomy and Dentistry Bldg, Montreal, QC H3A 0C7 Canada

**Keywords:** Single-cell repair, Plasma membrane repair, Cytoskeletal repair, Actin dynamics, Actomyosin ring, Live-confocal microscopy

## Abstract

**Objective:**

The contribution of actomyosin contractile rings in the wound healing program of somatic cells as never been directly assessed. This contrast with the events characterising the wound healing response of in wounded *Xenopus* oocytes, in which formation and contraction of an actomyosin ring provides a platform for cytoskeletal repair and drives the restoration of proper plasma membrane composition at the site of injury. As such, we aimed to characterize, using high-resolution live-cell confocal microscopy, the cytoskeletal repair dynamics of HeLa cells.

**Results:**

We confirm here that the F-actin enrichment that characterizes the late repair program of laser-wounded cells is mostly uniform and is not associated with co-enrichment of myosin-II or the formation of concentric zones of RhoA and Cdc42 activity.

**Electronic supplementary material:**

The online version of this article (10.1186/s13104-019-4441-7) contains supplementary material, which is available to authorized users.

## Introduction

Single-cell repair is highly context-dependent. Small, nanometer-sized plasma membrane (PM) wounds are thermodynamically favored to reseal spontaneously [1, 2]. Conversely, the repair of the very large wounds studied in *Xenopus laevis* and sea urchin oocytes, and in early drosophila embryos, requires the involvement of rapid exocytic events as well as the formation and “explodosis” of a large membrane patch [3]. In the same models, restoration of normal PM composition and of damaged cytoskeletal structures involves the cooperation between the contraction of an actomyosin ring [4] and actin polymerisation [5]. The repair of clinically and physiologically-relevant wounds experienced by somatic cells such as neurons, myofibers, and pneumonocytes also involves exocytic events are followed by the same core steps of wound stabilization: exocytosis of intracellular vesicles, followed by spontaneous repair, or the removal of damaged PM components by shedding or endocytosis (reviewed in [6]). It is also worth noting that none of these processes are able to fully restore a cell’s PM to its pre-wounded state [7], or restore damaged underlying cortex cytoskeletal structures [8, 9]. Somatic cells possess the necessary machinery to form contractile ring (CR) structures [10], but formation and contraction of a CR following PM injury have never been directly observed or confirmed. In *Xenopus* oocytes, CRs have been observed in response to puncture wounds of 150 µm in diameter [11] and laser wounds of approximately 50–100 µm in diameter [5, 12]. The wounds created in previous assays performed in mammalian cells are usually much smaller, historically in the range of a few μm in diameter. Is the smaller nature of the wounds created in somatic cells the reason for the apparent absence of a CR upon wounding?

We present here the results of a wound healing assay aiming to answer these questions and to directly investigate the presence of an CR following PM injury in somatic cells. Data was acquired by way of a laser-mediated wounding assay which created very large (10 μm range) wounds on the bottom surface of HeLa cells. We then imaged the PM and cytoskeletal repair dynamics using high-resolution, live confocal microscopy. We report strong microscopic evidence confirming that the wound healing response program of mammalian cells is mostly driven via actin polymerisation and does not necessarily involve the presence of a CR.

## Main text

### Materials and methods

#### Cell culture

HeLa cells purchased from ATCC (ATCC CCL-2) were maintained at 37 °C with 5% CO_2_, in MEM, supplemented with 10% FBS, 1% Penicillin–Streptomycin, and 1% Glutamax (all reagents from Gibco, Canada). For live imaging, cells were seeded on glass-bottom dishes (1.5 thickness; MatTek, USA) and imaged in phenol red-free imaging media.

#### PM labelling and targeted laser ablation of the PM

CellMask Deep Red Plasma Membrane Stain (Invitrogen, USA) was used to label the PM as per the manufacturer’s instructions and was used to target multiple sequential laser pulses of a MicroPoint UV laser (Andor, Northern Ireland) to a ~ 95 µm^2^ (11 µm diameter) area of the PM on the side contacting the dish (i.e. basal side). The success of PM ablation was made on the basis of the creation of a visually detectable gap in the fluorescent signal for both the PM (CellMask) and cytoskeletal compartments. Formation of bona fide PM and cytoskeletal wounds (vs photobleaching) was confirmed via a series of independent wounding/repair. FM1-43 (*N*-(3-Triethylammoniumpropyl)-4-(4-(Dibutylamino) Styryl) Pyridinium Dibromide; Invitrogen, USA) wounding assays [13] (Additional file 1), and re-assessed at regular intervals.

#### Transfection of genetically-encoded probes for live imaging

Cytoskeletal dynamics and RhoGTPase activity were tracked using a series of genetically encoded probes: F-actin (calponin homology domain of utrophin; PCS2-UtrCH-GFP; Addgene #26737 or PCS2-UtrCH-mCherry; Addgene #26740), myosin-II (RFP-tagged myosin regulatory light chain 1; PCS2-MRLC-RFP), microtubules (GFP-labelled microtubule binding-domain of ensconsin; PCS2-EMTB-3xGFP; Addgene # 26741), active RhoA (RhoA-binding domain of rhotekin; PCS2- γGBD-GFP; Addgene #26732), and Cdc42 (Cdc42 binding domain of N-WASP; PCS2- ωGBD-GFP Addgene # 26734). All plasmids were transfected using PolyJet In Vitro DNA transfection reagent (SignaGen, USA), as per the manufacturer’s instructions.

#### Imaging

Imaging was performed at the Cell Imaging and Analysis Network (CIAN) imaging facility at McGill University, using a Quorum WaveFX-X1 spinning disk confocal system on a Leica DMI6000B inverted microscope, two Hamamatsu “ImagEM X2 C9100-23B” EM-CCD cameras, and an 63×/1.40 oil objective (lBL, HCX PL APO; 506192). Cells were kept at 37 °C with 5% CO_2_ using a Live Cell Instruments Chamlide TC environmental control system. Acquisition was performed using MetaMorph Microscopy Automation and Image Analysis Software (Molecular Devices LLC, USA) and consisted of Z-stacks (11 slices, 0.2 µm apart), taken every 10 s for at least 15 min. 1 × 1 binning, 1 gain, and 100 EM gain was used throughout.

Images were further processed using ImageJ software (National Institutes of Health, USA). Quantitation of fluorescence at the wound site, was performed on maximum intensity projections (3 slices, 0.2 µm apart). Average fluorescence intensity was measured at every 10-s time point, at four 95 μm^2^ sites: the wound site region (FI_W_) and three control cytoplasmic regions within the cell (FI_C_). The relative fluorescence intensity at the wound site (FI_R_) was calculated by dividing FI_W_ by the average of the three FI_C_. To enable cell-to-cell comparisons, the resulting FI_R_ were then further normalized to the average FI_R_ on the same unwounded cell. Micrographs, kymographs, and orthogonal views were prepared from maximum intensity projections (MIPs) of the entire imaged volume (10 slices, 0.2 µm apart).

#### Statistics

Statistical analyses were performed using GraphPad Prism 7.04 (GraphPad Software Inc., USA) and consisted of repeated measure 2-way ANOVAs, followed by Dunnett’s multiple comparisons tests. Significance was set at p < 0.005.

### Results and discussion

We provide here substantial evidence that under normal 2D-culture conditions, mammalian cytoskeletal repair or large PM and cytoskeletal injuries involves actin polymerisation, without the formation of actomyosin contractile arrays.

#### Wound healing response involves uniform actin polymerisation, but not myosin-II accumulation

**Fig. 1:**
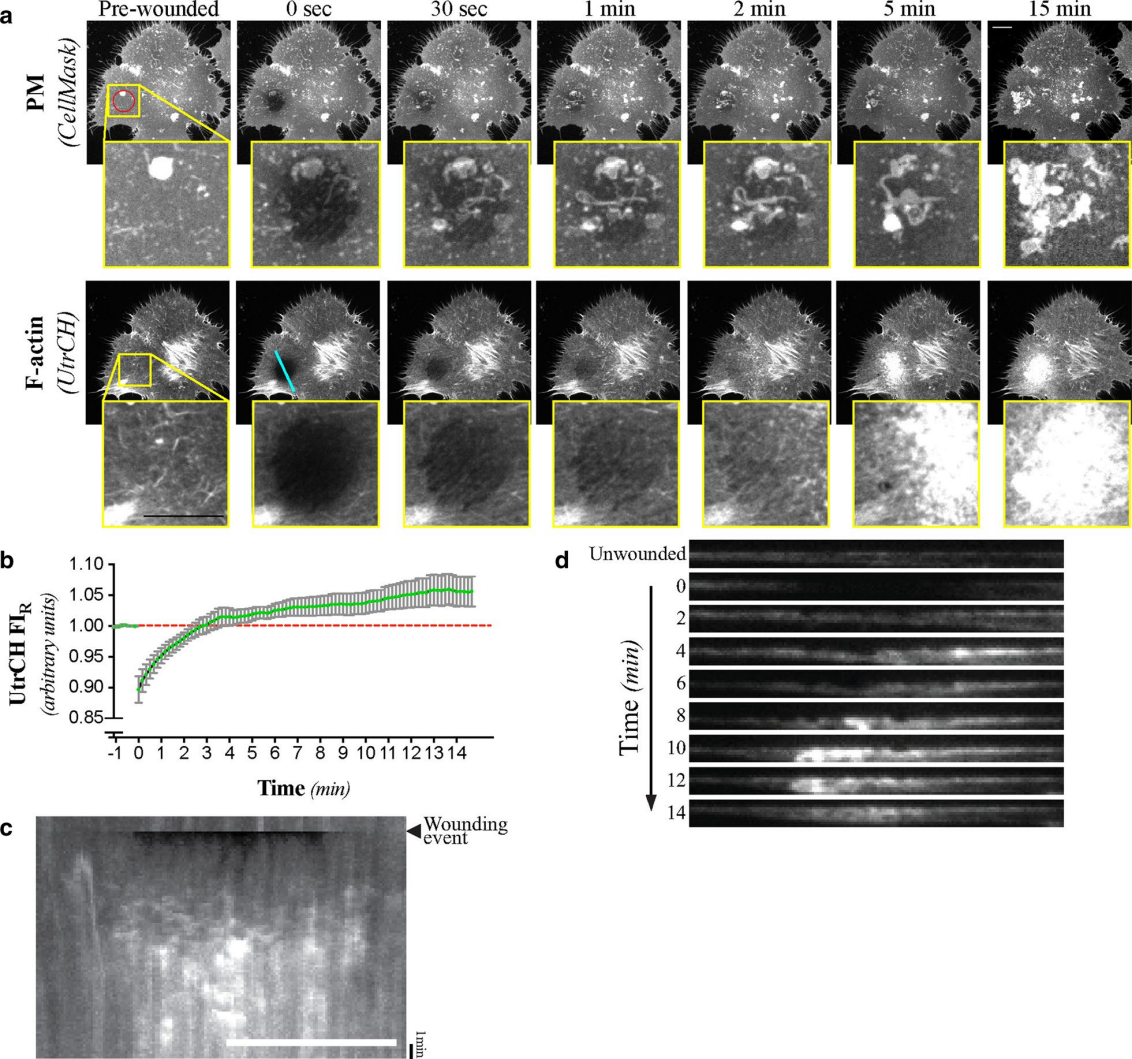
F-actin is enriched at the wound site following laser-mediated ablation of the PM. **a** Maximum intensity projections of selected micrographs of the CellMask and UtrCH signals following laser-mediated ablation of the PM of HeLa cells. **b** Normalized UtrCH fluorescent signal intensity (UtrCH FI_R_) at the wound site relative to control regions. Mean and SEM shown of n = 37 cells. **c** Kymograph of the UtrCH signal before and after laser-mediated ablation of the PM. Cell and targeted area is the same as in the one shown in **a**. **d** Resliced cortical volume (each 10 μm) of selected time-points of the wounding assay shown in **a**, **c**. Targeted area (95 μm^2^) is represented by the red circle found on the pre-wounded, PM micrograph. The line scan region used for the creation of the kymograph and resliced volumes correspond to the cyan line (22 μm) displayed on the 0 s, UtrCH micrograph. The cell shown in **a**, **c**, and **d** is representative of n = 37 wounding assays, all of which were used to prepare **b**. Scale bars = 10 μm

We first set out to also track the spatio-temporal dynamics of actin polymerisation that follows laser-mediated injury of HeLa cells. As expected, the ablated area showed clear indications of actin polymerisation following wounding (Figs. 1 and 3a; Additional file 2, Additional file 3, Additional file 4, Additional file 5, Additional file 6, Additional file 7, Additional file 8 and Additional file 9). Furthermore, UtrCH FI_R_ rapidly rose above the baseline post-wounded levels (significant after 30 s), which was significantly higher than the pre-wounding baseline levels after 6 min (Fig. 1b), suggesting enrichment of F-actin at the wound site. CR-mediated repair involves the formation of two functionally and structurally distinct zones of actin polymerisation: a dense ring of F-actin and Myosin-II located at the wound edge, which is surrounded by a wider array of perpendicularly oriented F-actin [5, 11, 14, 15]. Neither F-actin patterns were readily observable (Fig. 1a, 3a) during the course of more than 37 individual wounding assays. Kymographic and line scan analyses (Fig. 1c, 3b, c; Additional file 2) confirmed this qualitative assessment and also failed to reveal any other significant spatially defined enrichment of the UtrCH signal. Instead, UtrCH signal increased uniformly over the entire area of the wound site, with no noticeable enrichment at the wound edge (Fig. 1c, 3c; Additional file 2). Orthogonal views of the wound site were similarly unable to detect any localized UtrCH signal indicative of a clearly defined actomyosin structure (Fig. 1d).

**Fig. 2:**
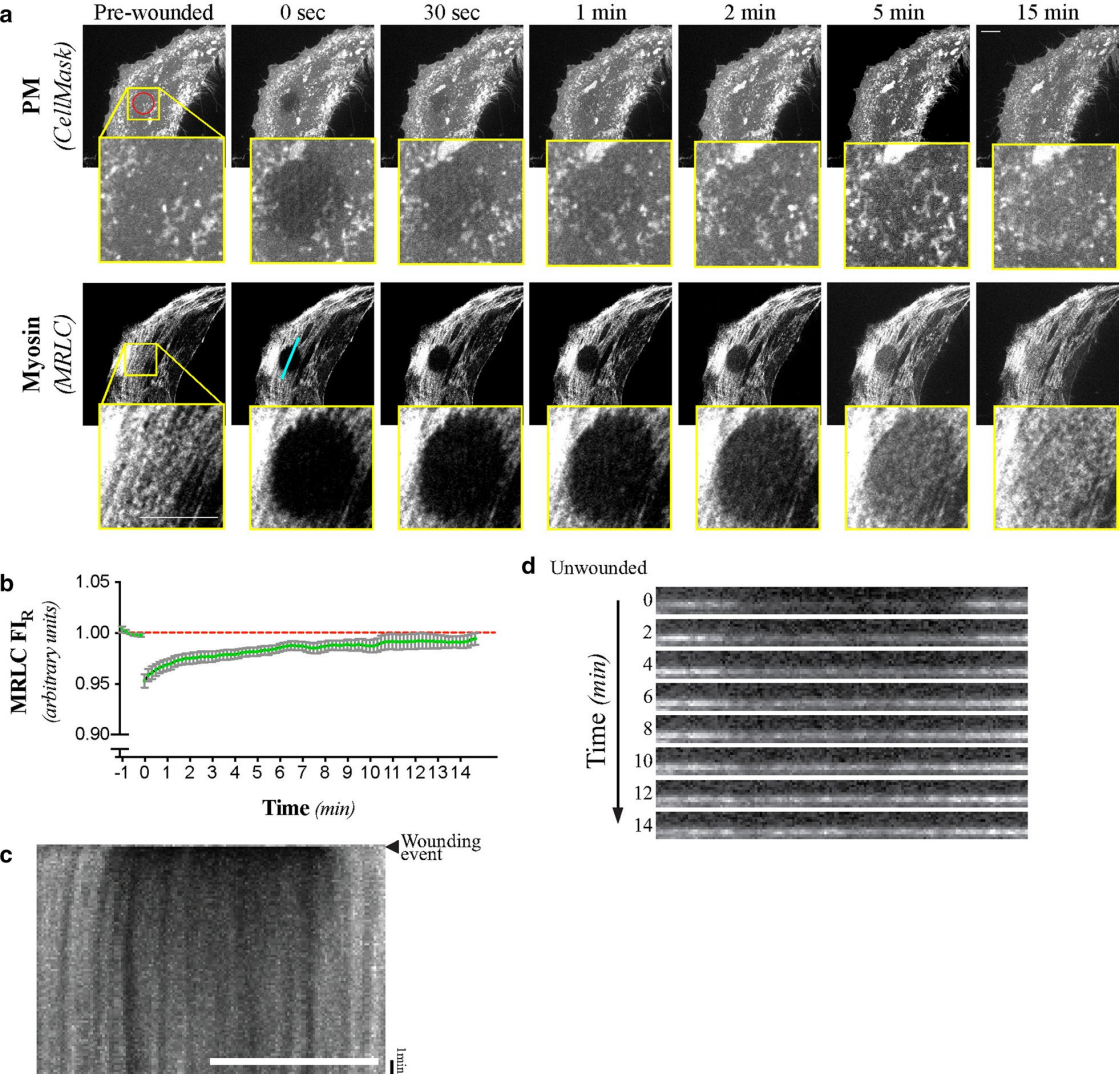
Mammalian single-cell repair does not involve myosin enrichment at the wound borders. **a** Maximum intensity projections of selected micrographs of the CellMask and MRLC signals following laser-mediated ablation of the PM of HeLa cells. **b** Normalized MLRC fluorescent signal intensity (MRLC FI_R_) at the wound site relative to control regions. Mean and SEM shown of n = 12 cells. **c** Kymograph of the MRLC signal before and after laser-mediated ablation of the PM. Cell and targeted area is the same as in the one shown in **a**. **d** Resliced cortical volume (each 10 μm) of selected time-points of the wounding assay shown in **a**, **c**. Targeted area (95 μm^2^) is represented by the red circle found on the pre-wounded, PM micrograph. The line scan region used for the creation of the kymograph and resliced volumes correspond to the cyan line (22 μm) displayed on the 0 s, MRLC micrograph. The cell shown in **a**, **c**, and **d** is representative of n = 12 wounding assays, all of which were used to prepare **b**. Scale bars = 10 μm

The spatio-temporal dynamics of myosin-II following injury was also investigated (Fig. 2a), as it is responsible for the actomyosin array’s contractility [11]. We found that the return of myosin-II at the wound site did not follow the same pattern as we observed for F-actin, as the rate of MRLC FI_RW_ signal accumulation to the wound site (Fig. 2b) was much slower than the one observed for UtrCH, and never went above pre-wounded baseline levels within the time allotted (Fig. 2b). Like F-actin, myosin-II did not show sign of accumulating according to any discernible patterns (Fig. 2a, c and d; Additional file 10). The sequence timing of F-actin and of myosin-II accumulation, broadly confirms the findings of other reports for wounds created on both the apical or basal side of cells, using a variety of wounding methods [16–18]. Specific differences in the actin polymerisation dynamics between our and all of the other previous studies could be attributable to the differences in wound size, wounding methods (laser vs mechanical), location (apical vs basal), cell shape and in local tension between the different wounding assays.

#### The mammalian wound healing response does not involve spatially regulated RhoA or Cdc42 signalling, microtubule arrays, or coordination of PM restoration and cytoskeletal repair

**Fig. 3:**
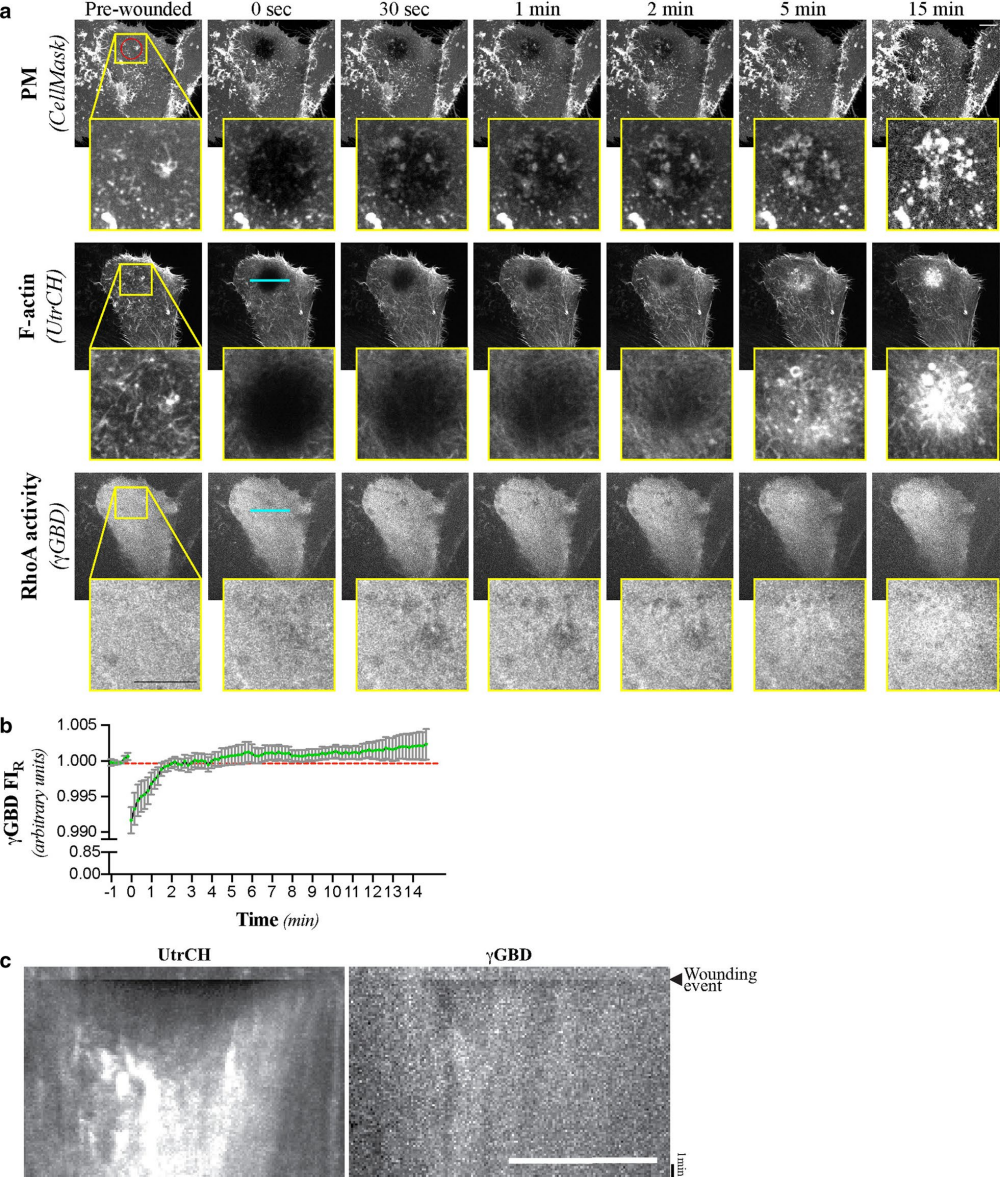
RhoA activity is increased the over entire area of the wound-site. **a** Maximum intensity projections of selected micrographs of the CellMask, UtrCH, and γGBD signals following laser-mediated ablation of the PM of HeLa cells. **b** Normalized γGBD fluorescent signal intensity (γGBD FI_R_) at the wound site relative to control regions. Mean and SEM shown of n = 12 cells. **c** Kymographs of the UtrCH and γGBD signals before and after laser-mediated ablation of the PM. Cell and targeted area is the same as in the one shown in **a**. Targeted area (95 μm^2^) is represented by the red circle found on the pre-wounded, PM micrograph. The line scan region used for the creation of the kymograph correspond to the cyan line (22 μm) displayed on the 0 s, γGBD micrograph. The cell shown in **a**, **c** is representative of n = 12 wounding assays, all of which were used to prepare **b**. Scale bars = 10 μm

The presence of concentric rings of RhoA and Cdc42 activity around the wound edge is a hallmark event of the formation of actomyosin contractile arrays [19]. As such, we set out to characterize the spatio-temporal distribution of RhoA and Cdc42 signalling in the context of our wound healing assay. Neither γGBD (Fig. 3) or ωGBD (Additional file 3) signals coalesced into the clearly defined RhoA or Cdc42 “activity zones” around the wound edge that would be necessary to the formation and contraction of CR, as demonstrated in wounded *Xenopus* oocytes [19, 20]. Instead, RhoA activity exhibited a pattern that closely matched that of UtrCH and accumulated over the entire area of the wound site (Fig. 3a, c). γGBD signal quickly rose after wounding, but never rose above its “pre-wounded” baseline levels (Fig. 3b). We were unable to observe any quantifiable or observable change in ωGBD following wounding anywhere in the cell, following the wounding event (Additional file 3; Additional file 7). These findings are consistent with a previous study that examined RhoA activity in mouse myoblasts wounded with a single laser blast to the basal side of their PM, which found local RhoA activation signal at the wound site that also failed to show ring-like patterning [18]. The same study attributed this pattern of RhoA activity to the increased reactive oxygen species (ROS) production caused by the increased mitochondrial uptake of calcium through the mitochondrial calcium uniporter (MCU) [18]. Whether mitochondria mediated ROS production also plays a role in the RhoA activation and actin polymerisation events observed in wounds as large as the ones produced within the context of our study remains to be confirmed.

We also failed to observe the formation of a MT array following injury. Instead, MTs seemed to polymerize from the wound-edge inward (Additional file  6; Additional file 11). This would be consistent with the absence of an actomyosin ring as CR formation is further driven by cortical flow and feedback with microtubules (MTs), which accumulate in a radial array around the wound site and the CR in the *Xenopus* oocyte model [5, 11, 12]. These observations are also compatible with a study of mouse myoblasts wounded with a single laser point, where MTs were also found to be neither enriched nor necessary for repair [18] and with observations about the dynamics of MTs of mechanically wounded fibroblasts [21].

In echinoderm oocytes, *Xenopus* oocytes, and early *Drosophila* embryos, PM and cytoskeletal repair are deeply interconnected (reviewed [6]). The contraction of the actomyosin array not only provides a platform for F-actin polymerisation, but also regenerates normal PM composition as it drags PM inwards through the contracting actomyosin array [14]. We did not observe such coordination in the healing rates of the PM and F-actin wounds (Additional file 12), which again suggests that cells from our wounding assay did not heal according to the classical model of actomyosin array-mediated contraction. It should be noted however, that our assays prioritized the visualization of cytoskeletal events, and as such did not set out to verify the presence of a de facto membrane patch as observable in the *Xenopus* model or of any of the exocytosis or “explocytosis” events that have been shown to be required for its formation [3].

### Conclusions

In mammalian somatic cells, repair of cytoskeletal wounds up to 10 μm is not strictly dependent on the assembly and contraction of an actomyosin array.

## Limitations

Apical and basal side of cells cultured in 2D probably exhibit differences in the properties of their cortex cytoskeleton structures or in their local tension. Exclusive targeting of the basal surface may have biased our results and limited compatibility with previously published mechanically wounded-cells or of the repair processes related to the apical surface.

## Additional files

**Additional file 1.** Rate of change of cytoplasmic FM1-43 fluorescence intensity (ΔFM1-43 cFI) in intact and wounded cells. In intact cells (doted light blue; n = 3), the rate of FM1-43 FI increase is very low and remains constant. In laser-ablated cells (solid green line; n = 10), the rate of FM1-43 FI is significantly increased following ablation, then returns to unwounded rates 20 s following ablation. Mean and SEM shown.

**Additional file 2.** Representative line scans of UtrCH FI at the wound sites of laser-ablated cells. A, C: Micrographs and associated kymographs of the UtrCH signal before and after laser-mediated ablation of the PM. B, D: Line-scans associated with the kymographs shown in A and C. Micrographs and kymographs are representative of n = 37 wounding assays.

**Additional file 3.** Laser-mediated ablation of the PM does not lead to increased Cdc42 activity. A: Maximum intensity projections of selected micrographs of the CellMask and ωGBD signals following laser-mediated ablation of the PM of HeLa cells. B: Normalized ωGBD fluorescent signal intensity (ωGBD FI_R_) at the wound site relative to control regions. Mean and SEM shown of n = 13 cells.

**Additional file 4.** Time-lapse video showing actin polymerisation at the site of laser-mediated injury. The video was prepared from maximum intensity projections of micrographs collected from 1 min before wounding, up to 15 min and 10 s after the wounding event, at a 10 s interval (98 micrographs/fluorescence channel) of the cell shown in Fig. 1. Signals corresponding the PM (cell mask) and F-actin (UtrCH) are shown to the left and right of the composite video, respectively

**Additional file 5.** Time-lapse video showing increased RhoA activity. The video was prepared from maximum intensity projections of micrographs collected 1 min before wounding, up to 15 min and 10 s after the wounding event, at a 10 s interval (98 micrographs/fluorescence channel) of the cell shown in Fig. 3. Signals corresponding the PM (cell mask), F-actin (UtrCH), and RhoA activity (γGBD) are shown to the left, center and right of the composite video, respectively.

**Additional file 6.** Time-lapse video showing microtubules growing inward from the wound-edge following laser-mediated injury. The video was prepared from maximum intensity projections of micrographs collected 30 s before wounding, up to 19 min and 20 s after the wounding event, at a 10 s interval (120 micrographs/fluorescence channel) of the cell shown in Additional file 11. Signals corresponding the PM (cell mask) and Microtubules (enconsin) are shown to the left and right of the composite video, respectively.

**Additional file 7.** Time-lapse video showing lack of noticeable Cdc42 activity following laser-mediated injury. The video was prepared from maximum intensity projections of micrographs collected 1 min before wounding, and up to 15 min and 10 s after the wounding event, at a 10 s interval (98 micrographs/fluorescence channel) of the cell shown in Additional file 3. Signals corresponding the PM (cell mask), F-actin (UtrCH), and Cdc42 activity (ωGBD) are shown to the left, center and right of the composite video, respectively.

**Additional file 8.** Time-lapse video of PM repair and actin polymerisation shown in Additional file 2A. The video was prepared from maximum intensity projections of micrographs collected from 1 min before wounding, up to 15 min and 10 s after the wounding event, at a 10 s interval (98 micrographs/fluorescence channel) of the cell shown in Additional file 2A. Signals corresponding the PM (cell mask) and F-actin (UtrCH) are shown to the left and right of the composite video, respectively.

**Additional file 9.** Time-lapse video of PM repair and actin polymerisation shown in Additional file 2B. The video was prepared from maximum intensity projections of micrographs collected from 1 min before wounding, up to 15 min and 10 s after the wounding event, at a 10 s interval (98 micrographs/fluorescence channel) of the cell shown in Additional file 2B. Signals corresponding the PM (cell mask) and F-actin (UtrCH) are shown to the left and right of the composite video, respectively.

**Additional file 10.** Time-lapse video showing myosin does not accumulate at the wound edges following laser-mediated injury. The video was prepared from maximum intensity projections of micrographs collected from 10 s before wounding, up to 20 min and 50 s after the wounding event, at a 10 s interval (127 micrographs /fluorescence channel) of the cell shown in Fig. 2. Signals corresponding the PM (cell mask) and myosin (MRLC) are shown to the left and right of the composite video, respectively.

**Additional file 11.** Microtubules grow inward from the wound-edge following laser-mediated ablation of the PM. A: Maximum intensity projections of selected micrographs of the CellMask and Enconsin signals following laser-mediated ablation of the PM of HeLa cells. B: Kymographs of the UtrCH and enconsin signals before and after laser-mediated ablation of the PM. C: Resliced cortical volume (each 10μm) of selected time-points of the wounding assay shown in A and C. Cell and targeted area is the same as in the one shown in A. Targeted area (95 μm^2^) is represented by the red circle found on the pre-wounded, PM micrograph. The line scan region used for the creation of the kymograph correspond to the cyan line (22μm) displayed on the 0 sec, F-actin micrograph. The cell shown in A and C is representative of n = 5. Scale bars = 10 μm.

**Additional file 12.** PM and Cytoskeletal response to PM wounding. A: The area of the dark region in the F-actin signal (solid light green line) is significantly bigger than the area of the dark region in the PM signal (dotted red line) for the first 60 s after wounding. Mean and SEM shown, n = 14 cells. B: The rate of change of the F-actin signal dark area (solid light green line) is significantly different from the rate of change of the PM signal dark area (dotted red line) across the fist 30 s post-ablation. Mean and SEM shown, n = 14 cells.

## Data Availability

Raw images used to produce the data are available from the corresponding author on reasonable request.

## Abbreviations

CR: contractile ring
Cdc42: cell division control protein 42 homolog
*Drosophila*: *Drosophila melanogaster*
F-actin: filamentous actin
PM: plasma membrane
MT: microtubule
MRLC: myosin regulatory light chain
N-WASP: neural Wiskott-Aldrich syndrome protein
RhoA: Ras homolog family member A
Rho GTPase: Rho family of GTP hydrolases
*Xenopus*: *Xenopus laevis*
