# Supplementary figures and images for: Dynamics of actin polymerisation during the mammalian single-cell wound healing response
Source: BMC Res Notes. 2019 Jul 16;12:420. doi: 10.1186/s13104-019-4441-7 (PMC6636100; doi:10.1186/s13104-019-4441-7)

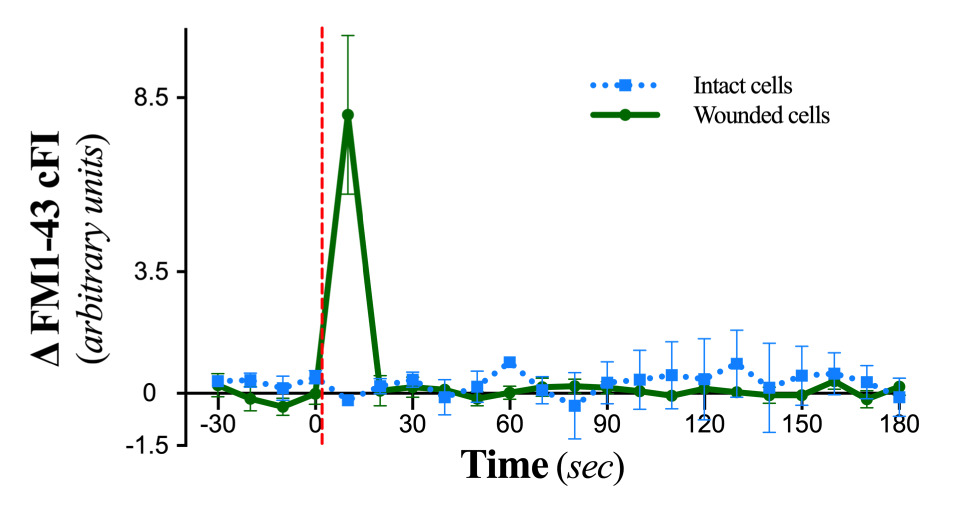

Supplement: Supplementary file 1 — Additional file 1. Rate of change of cytoplasmic FM1-43 fluorescence intensity (ΔFM1-43 cFI) in intact and wounded cells. In intact cells (doted light blue; n = 3), the rate of FM1-43 FI increase is very low and remains constant. In laser-ablated cells (solid green line; n = 10), the rate of FM1-43 FI is significantly increased following ablation, then returns to unwounded rates 20 s following ablation. Mean and SEM shown. [file 13104_2019_4441_MOESM1_ESM.tif]

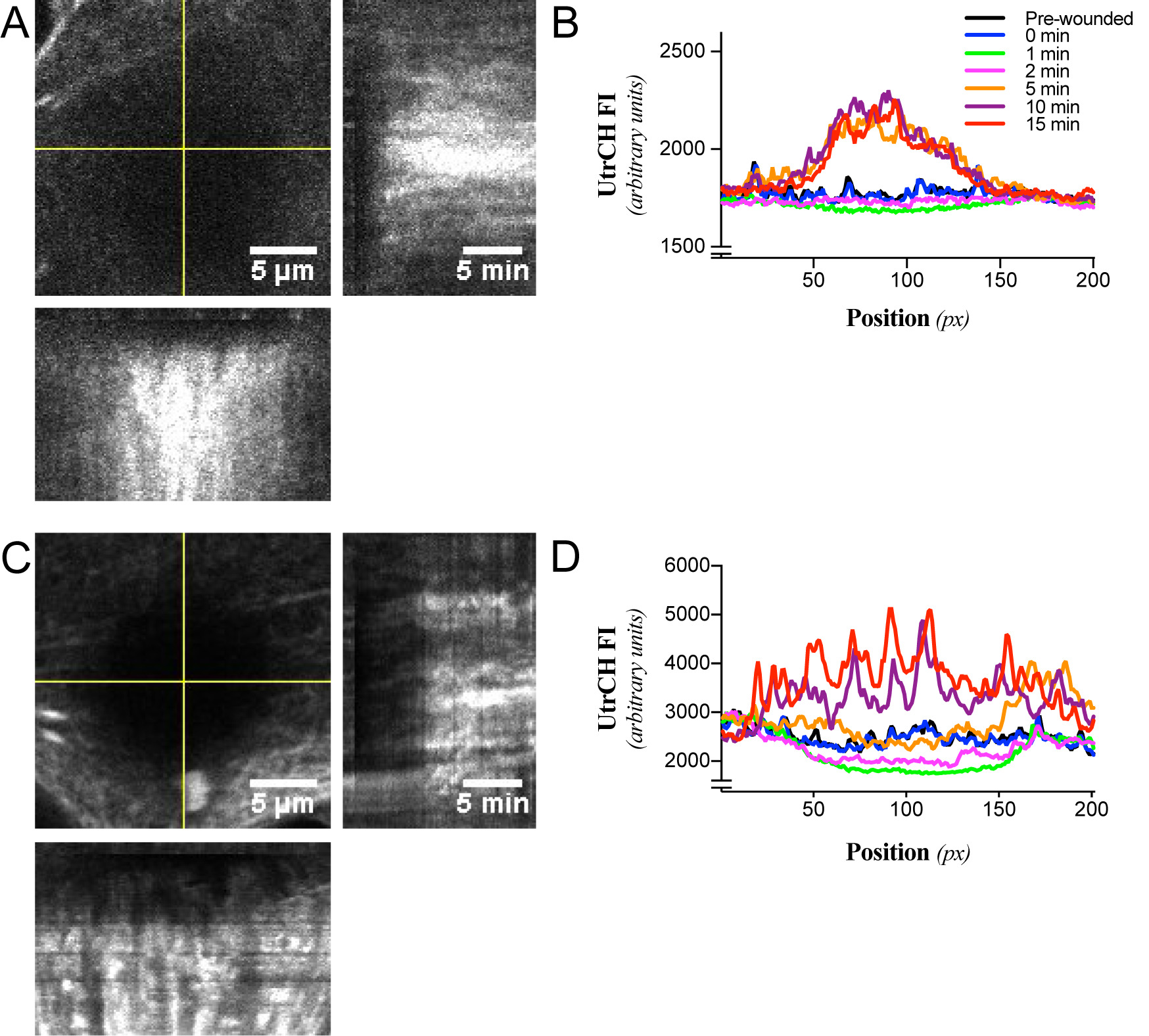

Supplement: Supplementary file 2 — Additional file 2. Representative line scans of UtrCH FI at the wound sites of laser-ablated cells. A, C: Micrographs and associated kymographs of the UtrCH signal before and after laser-mediated ablation of the PM. B, D: Line-scans associated with the kymographs shown in A and C. Micrographs and kymographs are representative of n = 37 wounding assays. [file 13104_2019_4441_MOESM2_ESM.tif]

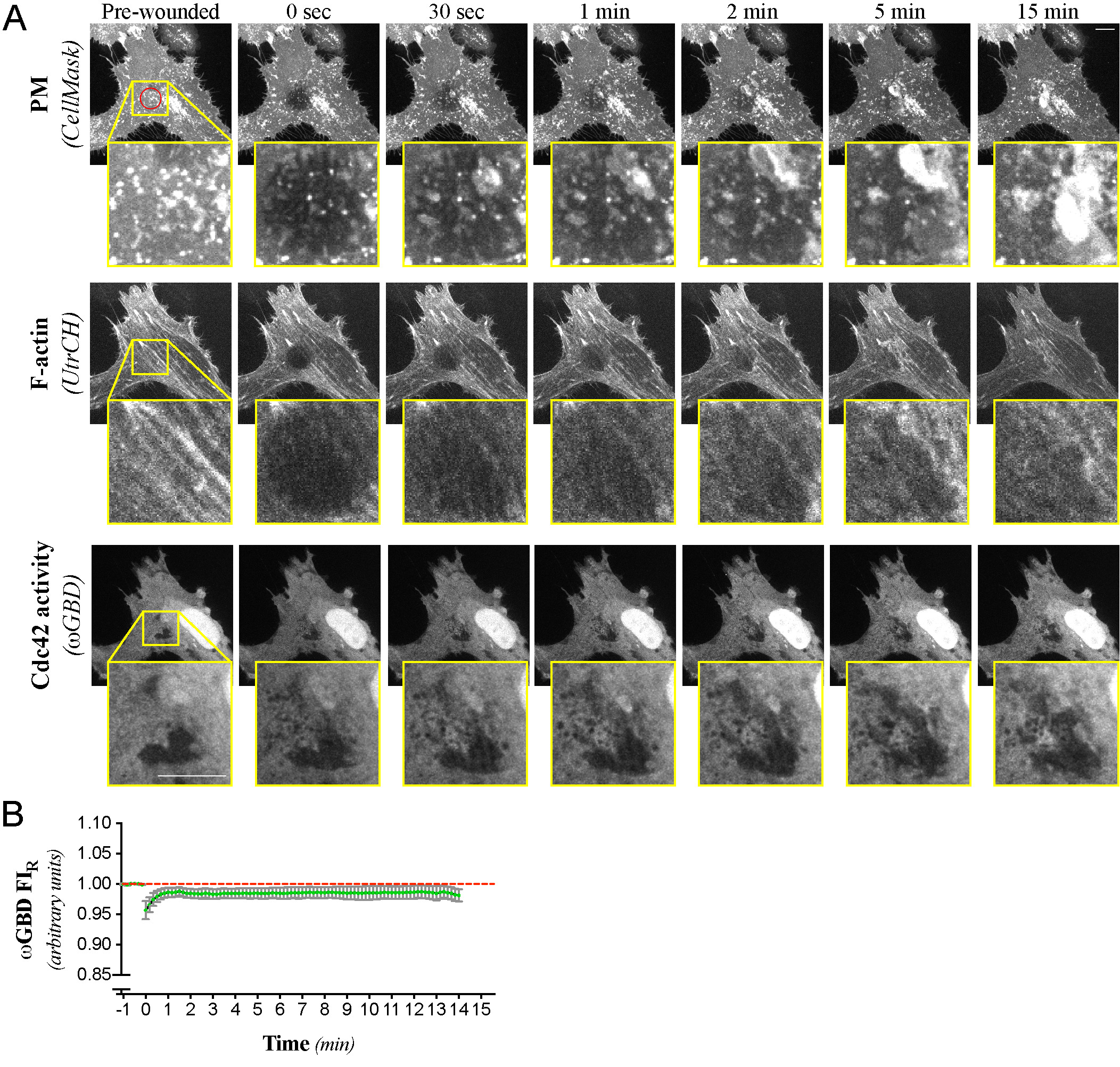

Supplement: Supplementary file 3 — Additional file 3. Laser-mediated ablation of the PM does not lead to increased Cdc42 activity. A: Maximum intensity projections of selected micrographs of the CellMask and ωGBD signals following laser-mediated ablation of the PM of HeLa cells. B: Normalized ωGBD fluorescent signal intensity (ωGBD FIR) at the wound site relative to control regions. Mean and SEM shown of n = 13 cells. [file 13104_2019_4441_MOESM3_ESM.tif]

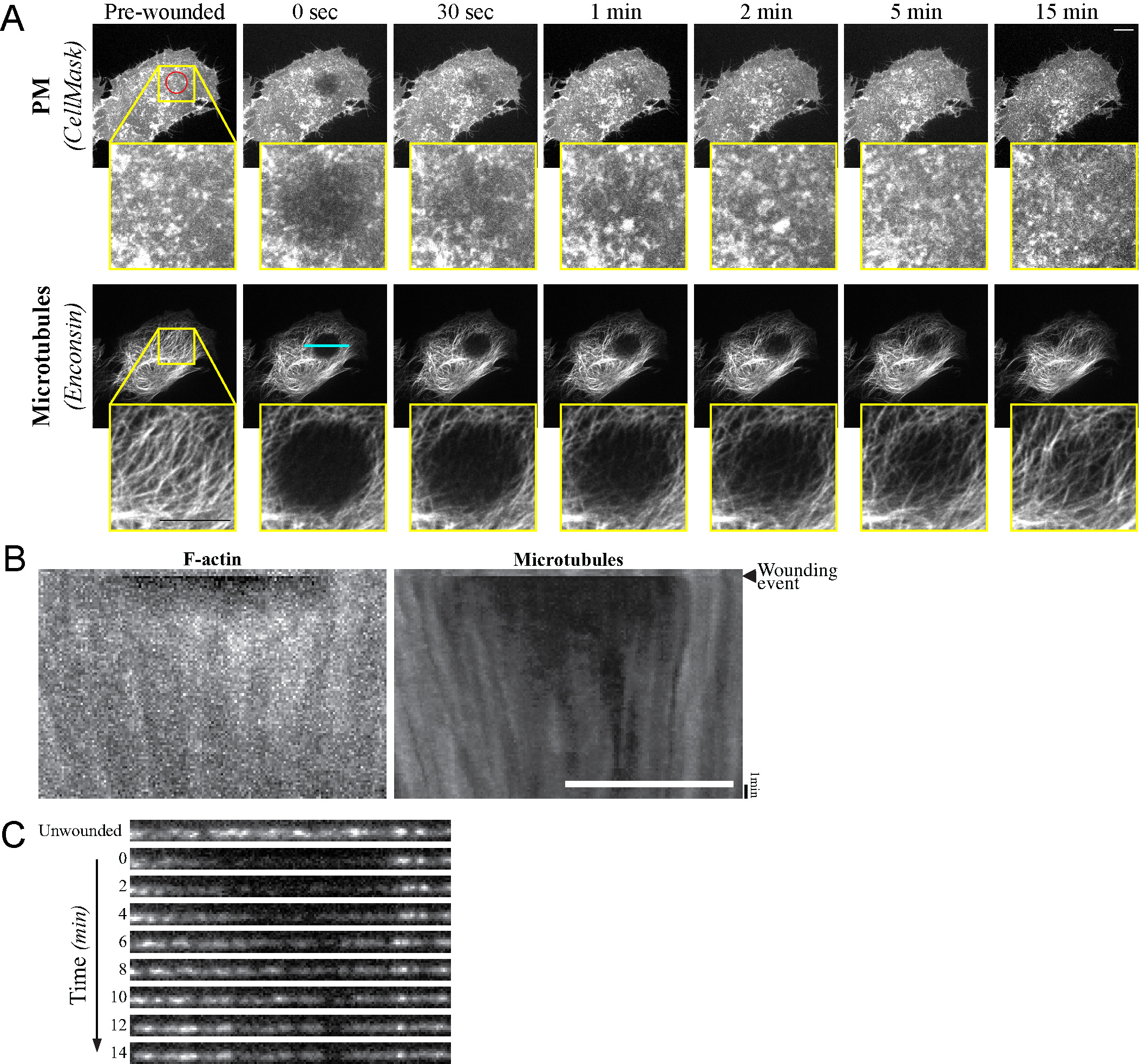

Supplement: Supplementary file 11 — Additional file 11. Microtubules grow inward from the wound-edge following laser-mediated ablation of the PM. A: Maximum intensity projections of selected micrographs of the CellMask and Enconsin signals following laser-mediated ablation of the PM of HeLa cells. B: Kymographs of the UtrCH and enconsin signals before and after laser-mediated ablation of the PM. C: Resliced cortical volume (each 10μm) of selected time-points of the wounding assay shown in A and C. Cell and targeted area is the same as in the one shown in A. Targeted area (95 μm2) is represented by the red circle found on the pre-wounded, PM micrograph. The line scan region used for the creation of the kymograph correspond to the cyan line (22μm) displayed on the 0 sec, F-actin micrograph. The cell shown in A and C is representative of n = 5. Scale bars = 10 μm. [file 13104_2019_4441_MOESM11_ESM.tif]

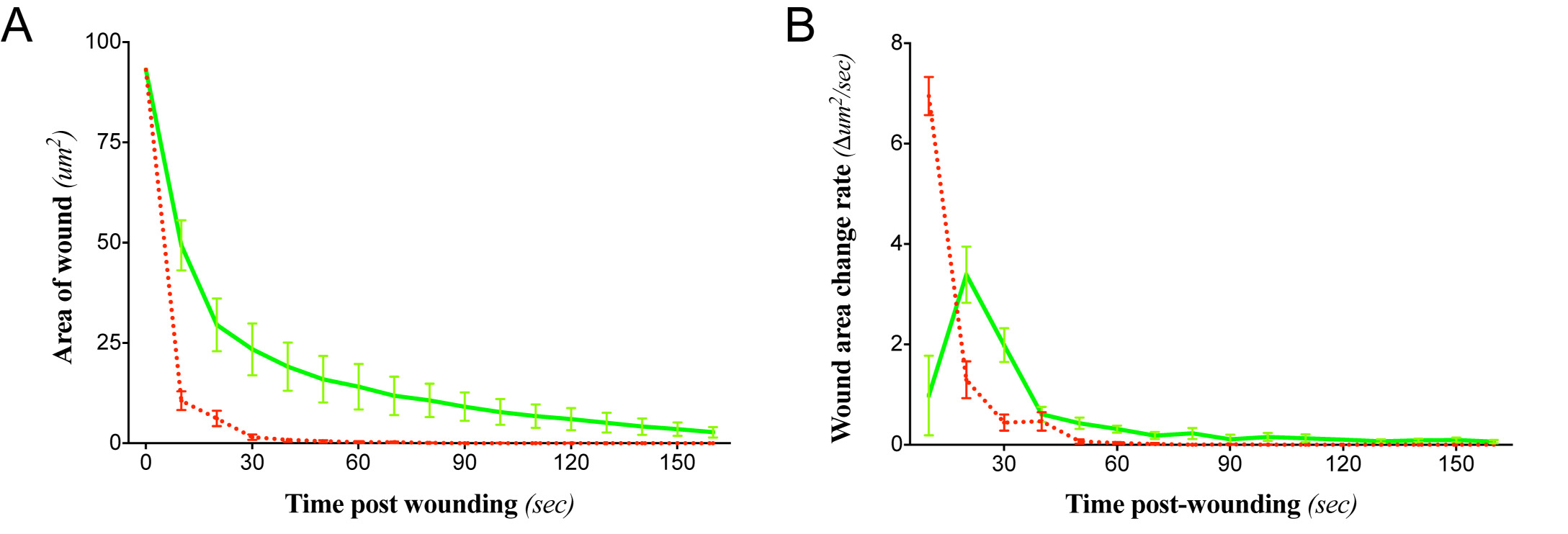

Supplement: Supplementary file 12 — Additional file 12. PM and Cytoskeletal response to PM wounding. A: The area of the dark region in the F-actin signal (solid light green line) is significantly bigger than the area of the dark region in the PM signal (dotted red line) for the first 60 s after wounding. Mean and SEM shown, n = 14 cells. B: The rate of change of the F-actin signal dark area (solid light green line) is significantly different from the rate of change of the PM signal dark area (dotted red line) across the fist 30 s post-ablation. Mean and SEM shown, n = 14 cells. [file 13104_2019_4441_MOESM12_ESM.tif]
